# DC-Derived IL-10 Modulates Pro-inflammatory Cytokine Production and Promotes Induction of CD4^+^IL-10^+^ Regulatory T Cells during *Plasmodium yoelii* Infection

**DOI:** 10.3389/fimmu.2017.00152

**Published:** 2017-02-28

**Authors:** Katharina Loevenich, Kristina Ueffing, Simone Abel, Matthias Hose, Kai Matuschewski, Astrid M. Westendorf, Jan Buer, Wiebke Hansen

**Affiliations:** ^1^Institute of Medical Microbiology, University Hospital Essen, University Duisburg-Essen, Essen, Germany; ^2^Institute of Biology, Humboldt University, Berlin, Germany; ^3^Parasitology Unit, Max Planck Institute for Infection Biology, Berlin, Germany

**Keywords:** rodent, parasitic protozoan, malaria, regulatory T cells, interleukin 10

## Abstract

The cytokine IL-10 plays a crucial role during malaria infection by counteracting the pro-inflammatory immune response. We and others demonstrated that *Plasmodium yoelii* infection results in enhanced IL-10 production in CD4^+^ T cells accompanied by the induction of an immunosuppressive phenotype. However, it is unclear whether this is a direct effect caused by the parasite or an indirect consequence due to T cell activation by IL-10-producing antigen-presenting cells. Here, we demonstrate that CD11c^+^CD11b^+^CD8^−^ dendritic cells (DCs) produce elevated levels of IL-10 after *P. yoelii* infection of BALB/c mice. DC-specific ablation of IL-10 in *P. yoelii*-infected IL-10^flox/flox^/CD11c-cre mice resulted in increased IFN-γ and TNF-α production with no effect on MHC-II, CD80, or CD86 expression in CD11c^+^ DCs. Accordingly, DC-specific ablation of IL-10 exacerbated systemic IFN-γ and IL-12 production without altering *P. yoelii* blood stage progression. Strikingly, DC-specific inactivation of IL-10 in *P. yoelii*-infected mice interfered with the induction of IL-10-producing CD4^+^ T cells while raising the frequency of IFN-γ-secreting CD4^+^ T cells. These results suggest that *P. yoelii* infection promotes IL-10 production in DCs, which in turn dampens secretion of pro-inflammatory cytokines and supports the induction of CD4^+^IL-10^+^ T cells.

## Introduction

Malaria is caused by the parasite *Plasmodium* and is one of the most important infectious diseases in humans worldwide. During blood stage propagation of *Plasmodium* infection, CD4^+^ T cells and humoral immune responses are essential components to control infection. However, immune responses against the parasite have to be tightly controlled to guarantee parasite clearance without excessive immune activation that might result in exacerbated tissue damage and increased mortality, emphasizing the need for immunoregulatory mechanisms (1, 2).

Recently, we demonstrated that infection of BALB/c mice with *Plasmodium yoelii* resulted in an expansion of naturally occurring CD4^+^Foxp3^+^ regulatory T cells (Tregs) (3, 4). Depletion of these cells by using the well-established DEREG mouse model (5) strongly increased T cell activation accompanied by a more efficient pathogen clearance (3). Besides Tregs, the cytokine IL-10 was identified as key immunoregulator during infection with different pathogens including *Plasmodium* ssp. (6). Injection of recombinant IL-10 protected susceptible mice from experimental cerebral malaria induced by *Plasmodium berghei* infection (7), and ablation of IL-10 resulted in higher plasma levels of pro-inflammatory cytokines and increased mortality of *Plasmodium chabaudi*-infected mice (8). IL-10-deficient mice infected with a non-lethal *P. yoelii* strain exhibited lower parasitemia (9), and ablation of IL-10 receptor (IL-10R) signaling resulted in decreased parasite burden in C57BL/6 mice infected with a virulent *P. yoelii* strain (10), underlining the importance of IL-10 in the regulation of immune responses during malaria infection.

It is well established that different immune cell types including B cells (11), macrophages, dendritic cells (DCs) (12), and several T cell subsets can produce IL-10 (13). During *P. yoelii* infection, we and other demonstrated elevated IL-10 expression by CD4^+^Foxp3^+^ Tregs and CD4^+^Foxp3^−^ T cells (3, 9). Induction of IL-10 production in CD4^+^Foxp3^−^ T cells during *P. yoelii* conferred immunosuppressive function to these cells, in terms of reduced proliferation and production of pro-inflammatory cytokines of cocultured responder cells (3). However, it remains unclear whether induction of these type 1 regulatory T cells (Tr1) is a parasite-driven effect or results from cytokine release of other immune cells and/or interaction with antigen-presenting cells (APCs), such as DCs, during the course of infection.

Dendritic cells are a heterogenous population of APCs playing a crucial role in the induction and regulation of cell-mediated immune responses (14). They can be subdivided into several subpopulations based on their expression of a variety of cell surface markers and their responses to pathogen molecules. Conventional CD11c^+^ dendritic cells mainly compromise of CD11b^−^CD8^+^ and CD11b^+^CD8^−^ subsets, which have been suggested to exhibit different functions. Whereas CD8^+^ DCs may have a superior ability to prime CD8^+^ T cells, CD11b^+^ DCs are thought to be more efficient in MHC-II antigen presentation to CD4^+^ T cells (15), the T cell type that plays an important role in the blood stage of malaria infection (16).

One immunoregulatory property of DCs is the production of IL-10 in the course of inflammatory responses (17–19) and infectious diseases (20–22). During parasitic infection ablation of IL-10R expression specifically in DCs resulted in enhanced immune responses associated with elevated Th1 responses and reduced parasitemia (23), comparable to ubiquitous IL-10-deficient mice (24), demonstrating that IL-10 might act in a paracrine and autocrine manner. IL-10 plays also an important role during interaction of DCs with T cells, since IL-10 signaling in DCs was suggested to be dispensable during naïve T cell priming but critical to prevent exaggerated effector T cell responses during skin inflammation (25). On the other hand, interaction of naïve T cells with IL-10-producing DCs was shown to induce immunosuppressive function (26–28). Since we and others detected the induction of IL-10-producing CD4^+^ Tr1 cells in the course of *P. yoelii* infection (3, 9), we asked whether DC-derived IL-10 contributes to this process. Therefore, we analyzed IL-10 secretion by DC subpopulations and its impact on the adaptive immune response in *P. yoelii*-infected mice.

## Materials and Methods

### Mice and Parasites

IL-10eGFP mice (29) (Jackson Laboratories, Bar Harbour, ME, USA), IL-10^flox/flox^ mice (30) (provided by Axel Roers, Dresden, Germany and Werner Müller, Manchester, UK), CD11c-cre mice (31), all on BALB/c background and BALB/c mice (Harlan Laboratories, Borchen, Germany) were crossed and maintained under specific pathogen-free conditions at the Animal Facility of the University Hospital Essen, Germany. Cryopreserved *P. yoelii* 17XNL (non-lethal)-infected red blood cells (iRBCs) were passaged once through BALB/c mice before being used in experimental animals. For infection 1 × 10^5^ iRBCs were injected i.v. The frequency of iRBCs (parasitemia) was determined by microscopic examination of Giemsa-stained blood films. The study was carried out in accordance with the guidelines of the German Animal Protection Law and the State Authority for Nature, Environment and Customer Protection, North Rhine-Westphalia, Germany. The protocol was approved by the State Authority for Nature, Environment and Customer Protection, North Rhine-Westphalia, Germany.

### Cell Isolation

Single cell suspensions of splenocytes were generated by rinsing spleens with erythrocyte lysis buffer and washing with PBS supplemented with 2% FCS and 2 mM EDTA. For the isolation of CD11c^+^ DCs, splenocytes were separated from contaminating superparamagnetic splenic red pulp cells (32) by using the AutoMACS Pro (Miltenyi Biotec, Bergisch Gladbach, Germany) before using MACS CD11c Microbeads (Miltenyi Biotec, Bergisch Gladbach, Germany) according to the manufacturer’s recommendations. CD4^+^ T cells were isolated from splenocytes using the CD4^+^ T cell isolation kit (Miltenyi Biotec, Bergisch Gladbach, Germany) according to the manufacturer’s protocol, followed by anti-CD4 staining and cell sorting using an Aria II Cell Sorter (BD Biosciences, Heidelberg, Germany).

### Antibodies and Flow Cytometry

Anti-CD4, anti-CD8, anti-CD11c, anti-CD11b, anti-MHC-II, anti-CD80, anti-CD86, anti-CD335, anti-CD3, anti-IFN-γ (all BD Biosciences, Heidelberg, Germany), anti-CD62L, anti-TNF-α, anti-Foxp3 (all eBioscience, Frankfurt, Germany), anti-CD160, anti-CD138, and anti-IL-10 (Biolegend, London, UK) were used as fluorescein isothiocyanate, pacific blue, phycoerythrin (PE), BD Horizon V450, allophycocyanin, AlexaFlour647, PE-cyanin 7, or peridinin-chlorophyll protein conjugates. Dead cells were identified by staining with the fixable viability dye eFlour 780 (eBioscience, Frankfurt, Germany). Intracelluar staining for Foxp3 was performed with the Foxp3 staining kit (eBioscience, Frankfurt, Germany) according to the manufacturer’s recommendations. Cytokine production from freshly isolated splenocytes was measured by stimulating cells with 10 ng/ml phorbol 12-myristate 13-acetate (PMA, Sigma-Aldrich, München, Germany) and 100 µg/ml ionomycin (Sigma-Aldrich, München, Germany) for 4 or 6 h (IL-10), respectively, in the presence of 5 µg/ml Brefeldin A (for IFN-γ, TNF-α staining) and 5 µg/ml Monensin (for IL-10 staining), treating with 2% paraformaldehyde and 0.1% NP40, and staining with the respective antibody cocktail. Flow cytometric expression analyses were performed with an LSR II instrument using DIVA software (BD Biosciences, Heidelberg, Germany).

### Serum Cytokine and IL-10 Production by DCs and CD4^+^ T Cells

Blood samples were collected, incubated at room temperature and centrifuged at 1,000 × g. Splenic CD11c^+^ DCs were stimulated overnight with 100 ng/ml liposaccharide (Sigma-Aldrich, München, Germany). Cytokines were quantified in the supernatants from stimulated DCs or CD4^+^ T cells, and in sera by using a Luminex Screening assay (R&D Systems, Wiesbaden, Germany) and a Luminex 200 system with Luminex IS software (Luminex Corporation, MV’s-Hertogenbosch, Netherlands) according to the manufacturer’s instructions. The detection limits were 1.32 pg/ml for IL-10, 0.73 pg/ml for IFN-γ, 0.13 pg/ml for TNF-α, and 1.31 pg/ml for IL-12, respectively.

### Statistical Analysis

Statistical analyses were performed with one-way ANOVA, Student’s *t*-test for parametric and Mann–Whitney test for non-parametric distributed data as indicated with significance set at the levels of *p < 0.05, **p < 0.01, and ***p < 0.001. Normality was tested using the D’Agostino–Pearson omnibus and Kolmogorov–Smirnov test. All analyses were calculated with Graph Pad Prism 5.0 Software (Graph Pad Software, La Jolla, CA, USA).

## Results

### Enhanced Frequencies of IL-10-Producing CD11c^+^CD11b^+^CD8^−^ DCs in *P. yoelii*-Infected Mice

**Figure 1:**
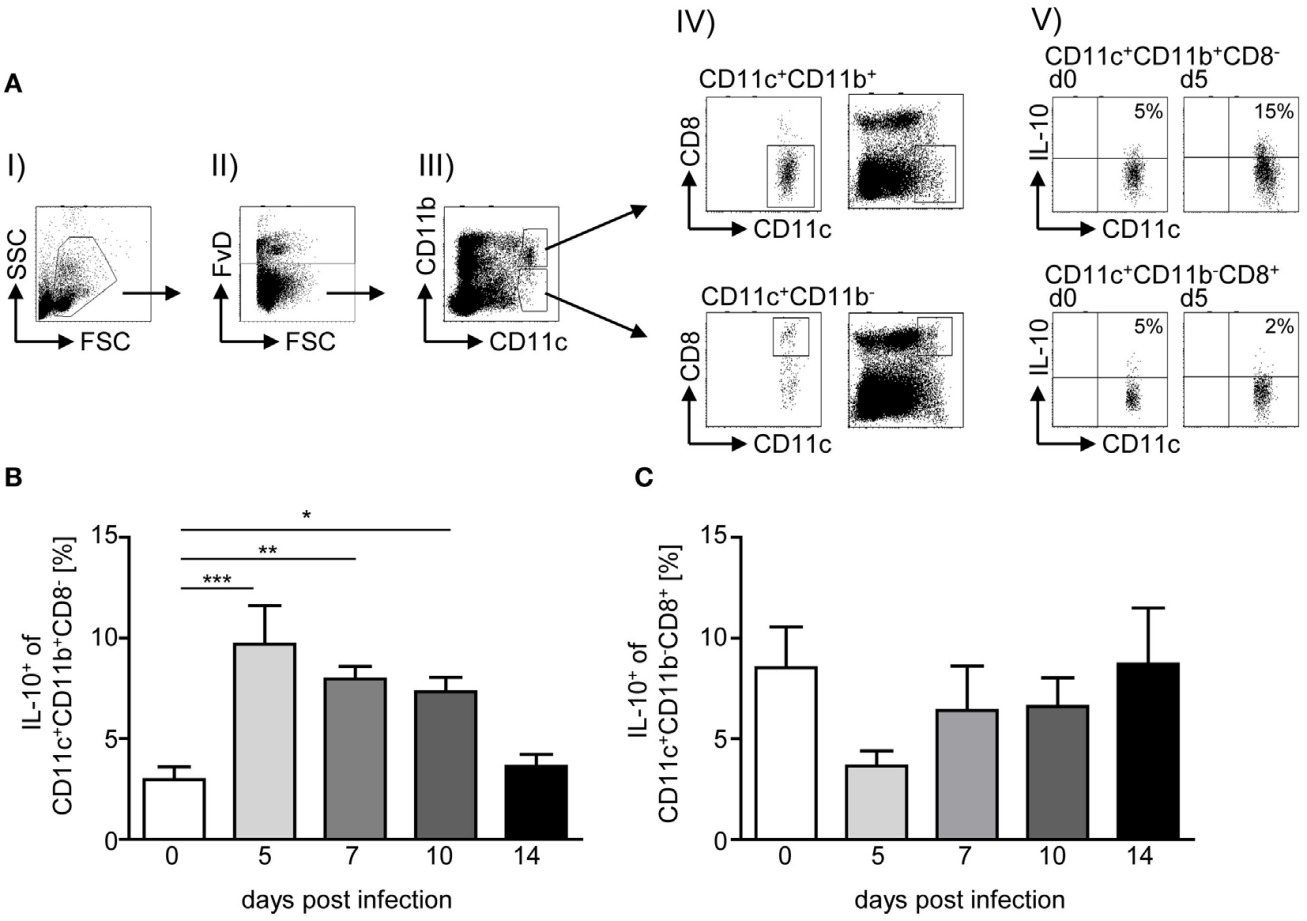
**Elevated IL-10 production of CD11c^+^CD11b^+^CD8^−^ dendritic cells (DCs) upon *Plasmodium yoelii* infection**. IL-10eGFP reporter mice were infected with infected red blood cells at day 0. At day 5, 7, 10, and 14 postinfection (p.i.), the percentages of IL-10-expressing (eGFP^+^) **(B)** CD11c^+^CD11b^+^CD8^−^ DCs and **(C)** CD11c^+^CD11b^−^CD8^+^ DCs were determined by flow cytometry. **(A)** Representative dot plots illustrate the gating strategy: (I) selection of mononuclear cells, (II) gating on viable cells, (III) gating on CD11c^+^CD11b^+^ and CD11c^+^CD11b^−^ cells, respectively, and (IV) selection of CD8^−^ from gated CD11c^+^CD11b^+^ (upper panel) and CD8^+^ from gated CD11c^+^CD11b^−^ cells (lower panel). Additionally, we depicted all viable cells together with gated CD8^−^ from CD11c^+^CD11b^+^ and CD8^+^ from gated CD11c^+^CD11b^−^ cells, respectively (right panel). (V) Representative dot plots showing IL-10-expressing CD11c^+^ subsets as indicated. Data from *n* = 6–12 mice per time point out of at least two individual experiments are summarized as mean ± SEM. One-way ANOVA with Dunnett’s posttest was used for statistical analysis (*p < 0.05, **p < 0.01, ***p < 0.001).

IL-10 plays an important role in the regulation of *P. yoelii* infection, and CD4^+^ T cells have been shown to secrete high amounts of IL-10 upon infection (3, 9). To gain further insights into the question whether IL-10 induction in CD4^+^ T cells is a direct effect of the parasite or results from IL-10 secretion by APCs, we analyzed the frequency of IL-10-producing (eGFP^+^) CD11c^+^CD11b^+^CD8^−^ DCs and CD11c^+^CD11b^−^CD8^+^ DCs in spleen of IL-10eGFP reporter mice at different time points post-*P. yoelii* infection by flow cytometry (Figure 1A). Whereas we observed significantly elevated percentages of IL-10-expressing CD11c^+^CD11b^+^CD8^−^ DCs in the course of infection (Figure 1B), the frequency of IL-10^+^ CD11c^+^CD11b^−^CD8^+^ DCs was reduced at day 5 postinfection but recovered to levels of non-infected mice at day 7, 10, and 14 postinfection (Figure 1C).

### DC-Specific IL-10 Inactivation Results in Elevated Production of Pro-inflammatory Cytokines by CD11c^+^ DCs during *P. yoelii* Infection

**Figure 2:**
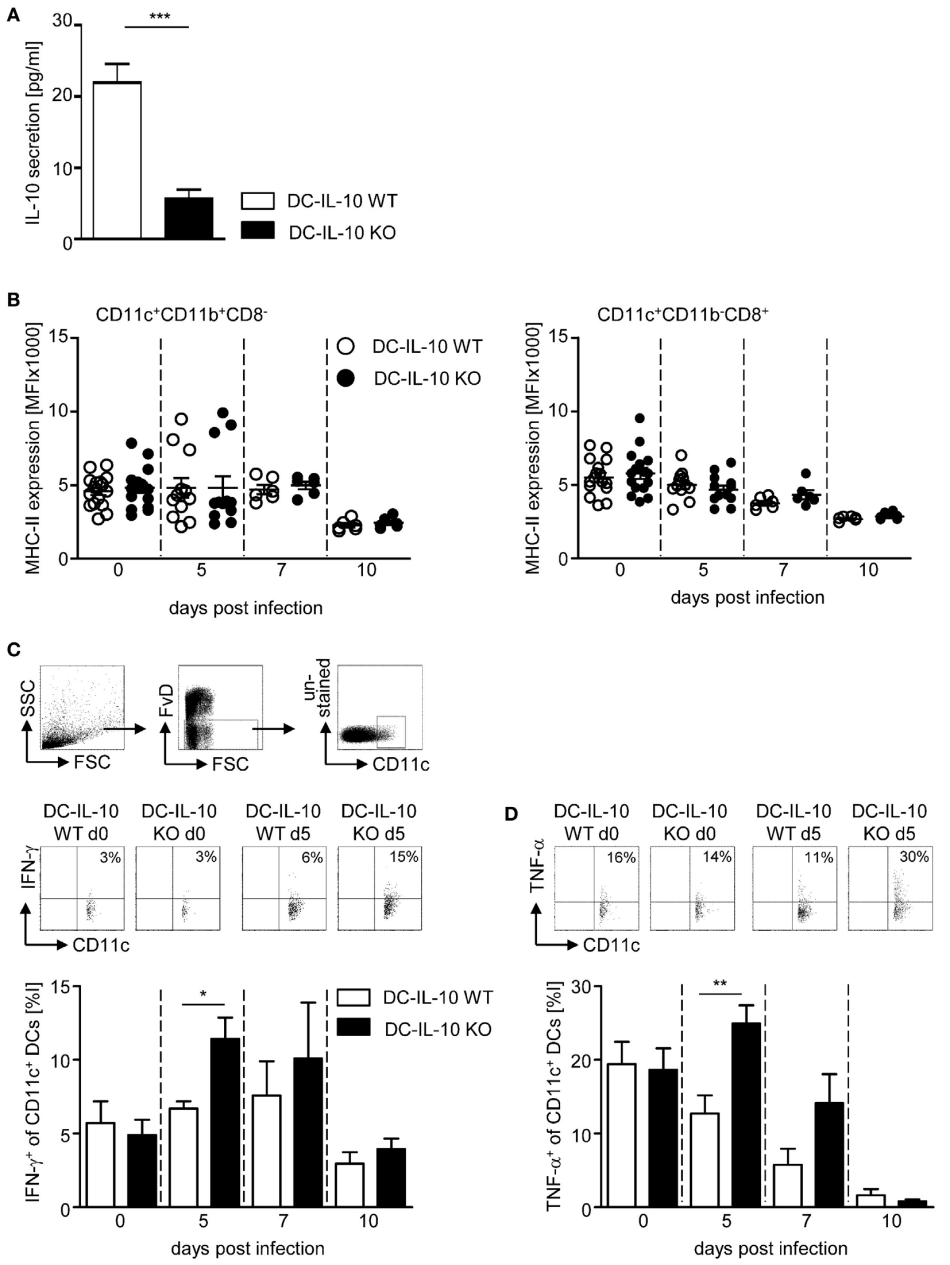
**Inactivation of IL-10 in IL-10^flox/flox^/CD11c-cre mice results in enhanced IFN-γ and TNF-α production of CD11c^+^ dendritic cells (DCs) during *Plasmodium yoelii* infection**. **(A)** IL-10 secretion of LPS-stimulated CD11c^+^ DCs isolated from spleen of IL-10^flox/flox^ (DC-IL-10 WT) and IL-10^flox/flox^/CD11c-cre (DC-IL-10 KO) was analyzed by Luminex technology. IL-10^flox/flox^ and IL-10^flox/flox^/CD11c-cre mice were infected with *P. yoelii*. At indicated time points postinfection, **(B)** the expression level (MFI) of MHC-II was analyzed on gated CD11c^+^CD11b^+^CD8^−^ DCs and CD11c^+^CD11b^−^CD8^+^ DCs, and the **(C)** frequencies of IFN-γ and **(D)** TNF-α-expressing CD11c^+^ DCs were determined by flow cytometry. The gating strategy of CD11c^+^ DCs and representative dot plots are shown in the upper panel. Results from at least two independent experiments with *n* = 6–16 mice per time point were summarized as mean ± SEM. Student’s *t*-test was used for statistical analysis (*p < 0.05, **p < 0.01, ***p < 0.01).

To investigate the impact of DC-derived IL-10 on the immune response during *P. yoelii* infection of BALB/c mice, we made use of IL-10^flox/flox^/CD11c-cre mice to specifically ablate IL-10 expression in CD11c^+^ DCs. To confirm functional inactivation of IL-10, we isolated CD11c^+^ DCs from IL-10^flox/flox^/CD11c-cre mice and IL-10^flox/flox^ littermates, stimulated them *in vitro* with LPS and analyzed the amount of IL-10 in the supernatant by Luminex technology. As depicted in Figure 2A, CD11c^+^ DCs from IL-10^flox/flox^ control mice secreted approximately 22 pg/ml IL-10 to the culture medium, whereas cells from DC-specific IL-10-deficient (IL-10^flox/flox^/CD11c-cre) mice produced significantly less IL-10 upon LPS stimulation (approximately 5 pg/ml). Hence, the IL-10^flox/flox^/CD11c-cre mouse line is a suitable model to investigate the impact of DC-derived IL-10 on the course of *P. yoelii* infection.

We infected DC-specific IL-10-deficient mice and WT littermates with *P. yoelii* and analyzed the expression of MHC-II on CD11c^+^CD11b^+^CD8^−^ DCs and CD11c^+^CD11b^−^CD8^+^ DCs at different time points postinfection by flow cytometry. We observed only a slight, albeit non-significant, reduction in MHC-II expression levels in the course of infection on both DC subtypes, and this was irrespective of their IL-10 expression (Figure 2B). Similarly, CD80 and CD86 expression on DCs slightly decreased at later time points postinfection, but again with no significant differences between IL-10-deficient and WT DCs (Figures S1A,B in Supplementary Material). However, significant higher percentages of CD11c^+^ DCs from IL-10^flox/flox^/CD11c-cre mice produced IFN-γ and TNF-α at early time points after *P. yoelii* infection in comparison to WT controls (Figures 2C,D). To exclude contaminating NK, NKT, and T cells within the CD11c^+^ DC population, we extended our analysis for IFN-γ production to CD335^−^CD3^−^CD160^−^CD11c^+^ DCs from non-infected and *P. yoelii*-infected mice at day 5 postinfection and obtained same results, showing higher percentages of IFN-γ-expressing CD335^−^CD3^−^CD160^−^CD11c^+^ DCs from IL-10^flox/flox^/CD11c-cre mice than from control mice (Figure S2 in Supplementary Material). These results suggest that DC-derived IL-10 has no impact on the capability of DCs to present antigen and on their costimulatory activity, but transiently alters the cytokine profile of CD11c^+^ DCs during *P. yoelii* infection.

### Increased IFN-γ Expression in CD4^+^ T Cells from DC-Specific IL-10-Deficient *P. yoelii*-Infected Mice

**Figure 3:**
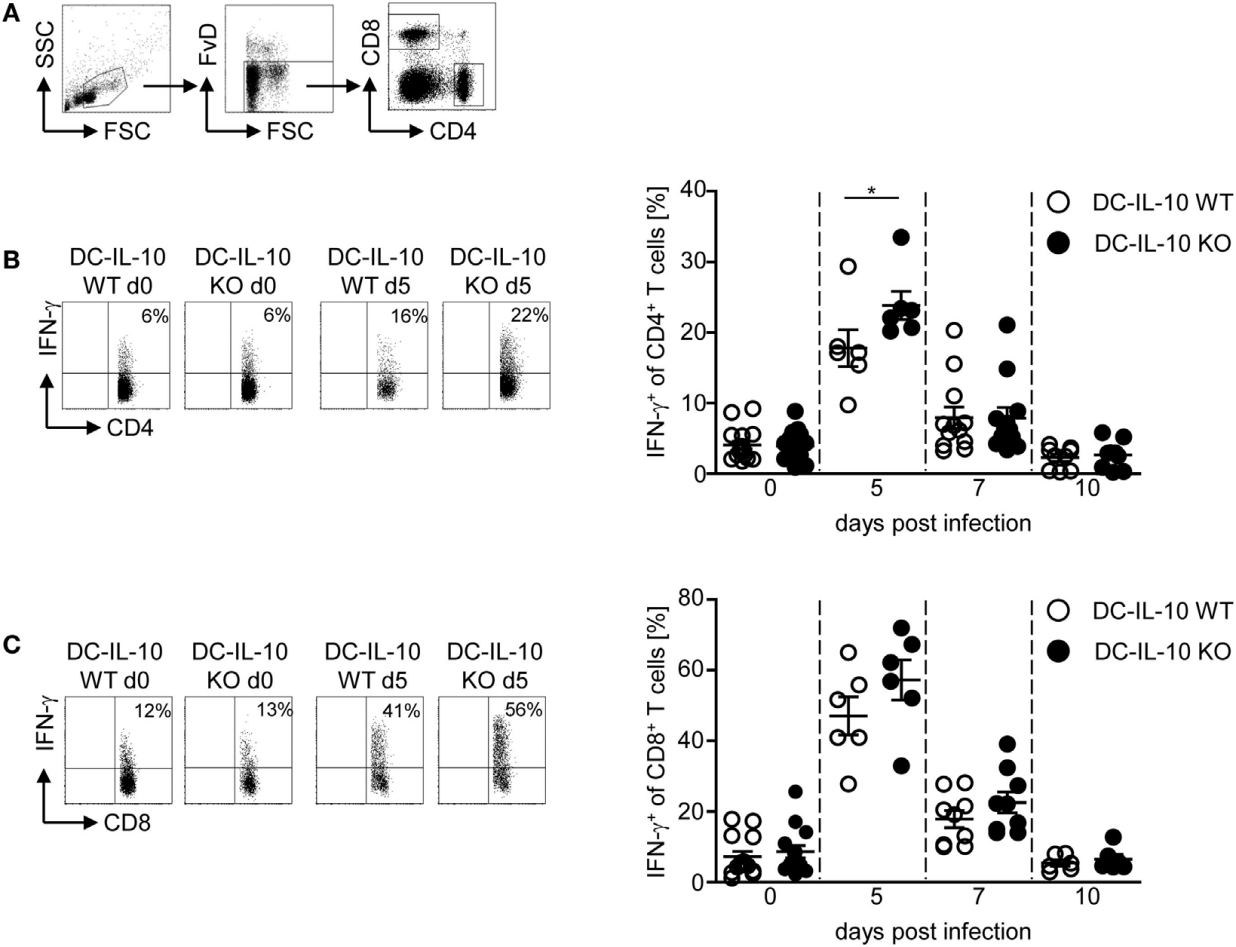
**Increase of IFN-γ-producing CD4^+^ T cells in *Plasmodium yoelii*-infected IL-10^flox/flox^/CD11c-cre mice**. **(A)** Representative gating strategy for CD4^+^ and CD8^+^ T cells. The frequency of IFN-γ-producing **(B)** CD4^+^ and **(C)** CD8^+^ T cells from non-infected and *P. yoelii*-infected IL-10^flox/flox^ [dendritic cells (DC)-IL-10 WT] and IL-10^flox/flox^/CD11c-cre (DC-IL-10 KO) mice at day 5, 7, and 10 p.i. was determined by intracellular staining and flow cytometric analysis. Representative dot plots are shown in the left panel. Data from two to three independent experiments with *n* = 6–16 mice per time point are depicted as mean ± SEM. Mann–Whitney test was used for statistical analysis (*p < 0.05).

Next, we asked whether the altered cytokine profile of IL-10-deficient CD11c^+^ DCs during *P. yoelii* infection has an impact on the T cell response. For this purpose, we infected IL-10^flox/flox^/CD11c-cre and IL-10^flox/flox^ control mice with *P. yoelii* and determined the frequency of CD62L-expressing CD4^+^ and CD8^+^ T cells. As expected, the percentage of CD62L-expressing T cells was reduced in *P. yoelii*-infected mice, but we detected no differences between mice with IL-10-deficient CD11c^+^ DCs and WT littermates (Figures S3A,B in Supplementary Material). However, functional inactivation of IL-10 in CD11c^+^ DCs resulted in significantly elevated IFN-γ production in CD4^+^ T cells (Figures 3A,B) and slightly, but not significantly, increased frequencies of IFN-γ-expressing CD8^+^ T cells (Figures 3A,C) at day 5 post-*P. yoelii* infection. Hence, inactivation of IL-10 in CD11c^+^ DCs does not impede T cell activation, but rather induces IFN-γ production in CD4^+^ T cells during *P. yoelii* infection.

### DC-Derived IL-10 Promotes the Induction of IL-10-Producing CD4^+^ Tr1 Cells in *P. yoelii*-Infected Mice with Normal Parasite Propagation

**Figure 4:**
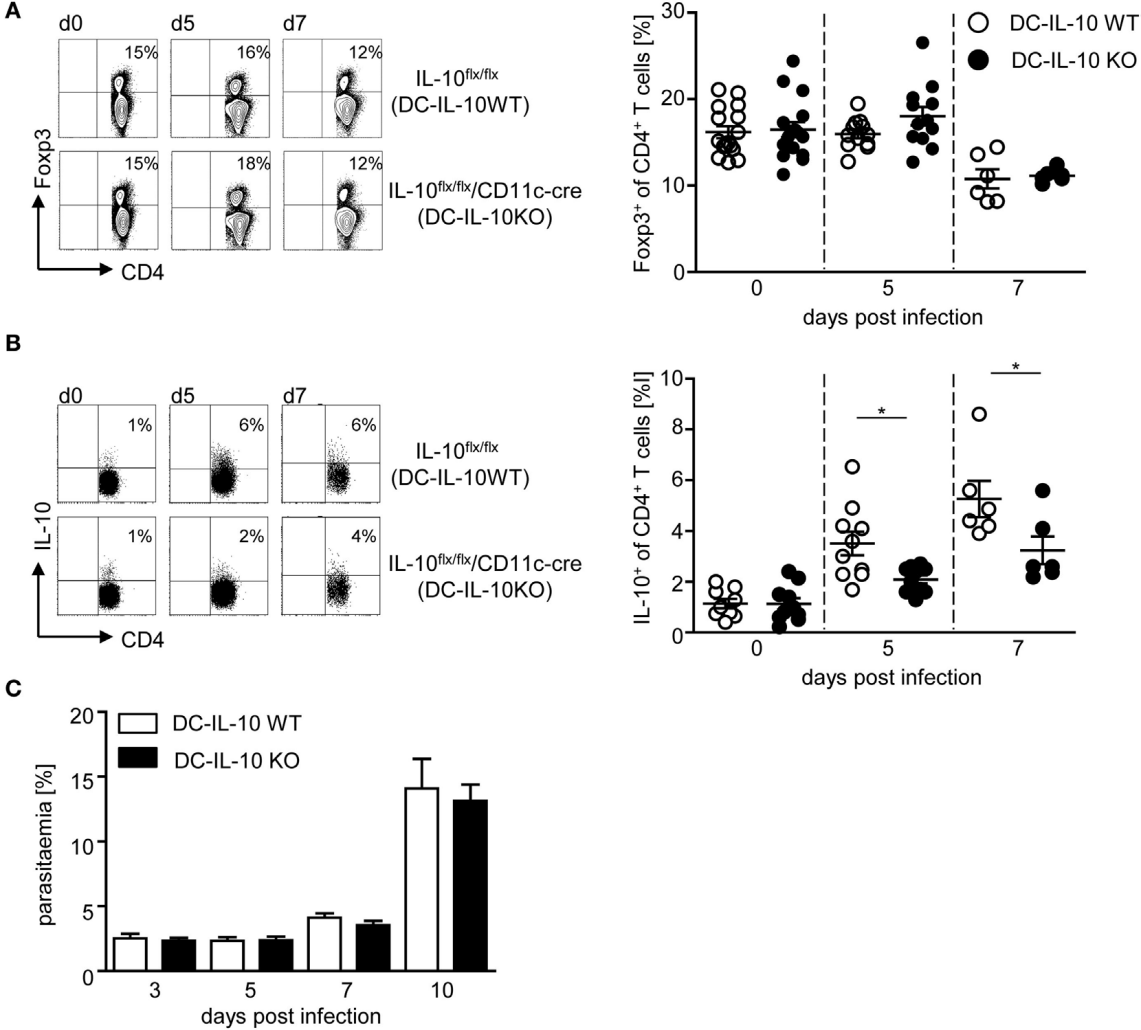
**Impaired induction of IL-10-expressing CD4^+^ type 1 regulatory T cells and normal parasite propagation in *Plasmodium yoelii*-infected mice with IL-10-deficient CD11c^+^ dendritic cells (DCs)**. Percentages of **(A)** Foxp3 and **(B)** IL-10-expressing CD4^+^ T cells were measured by flow cytometry in non-infected and *P. yoelii*-infected IL-10^flox/flox^ (DC-IL-10 WT) and IL-10^flox/flox^/CD11c-cre (DC-IL-10 KO) mice at indicated time points p.i. Representative dot plots are depicted in the left panels. **(C)** Parasitemia of *P. yoelii*-infected IL-10^flox/flox^ (DC-IL-10 WT) and IL-10^flox/flox^/CD11c-cre (DC-IL-10 KO) mice was determined by Giemsa staining. Results from at least two independent experiments with *n* = 6–16 mice **(A,B)** and *n* = 18–32 mice **(C)** per time point are summarized as mean ± SEM. Mann–Whitney test was used for statistical analysis (*p < 0.05).

To determine whether DC-derived IL-10 has an impact on CD4^+^ Treg subsets, we next analyzed the effect of DC-specific IL-10 ablation during *P. yoelii* infection on the amount of Foxp3^+^ Tregs and IL-10^+^ Tr1 cells. As depicted in Figure 4A, flow cytometric analyses revealed no differences in the frequency of Foxp3^+^ Tregs from *P. yoelii*-infected IL-10^flox/flox^/CD11c-cre mice in comparison to IL-10^flox/flox^ littermates (Figure 4A). Strikingly, we observed a significant lower induction of IL-10-producing CD4^+^ Tr1 cells in IL-10^flox/flox^/CD11c-cre mice than in IL-10^flox/flox^ control mice at day 5 and 7 post-*P. yoelii* infection (Figure 4B). To exclude, that IL-10 expression in CD4^+^ T cells is affected by CD11c-driven cre expression, we analyzed IL-10 secretion of sorted CD4^+^ T cells from naive IL-10^flox/flox^/CD11c-cre mice and IL-10^flox/flox^ control mice after stimulation *in vitro* by Luminex and detected no significant differences (Figure S4A in Supplementary Material). Moreover, CD4^+^ T cells from both DC-specific IL-10-deficient and control mice produced similar amounts of IFN-γ and TNF-α after stimulation *in vitro* (Figures S4B,C in Supplementary Material), indicating that CD11c-driven cre expression has no influence on IL-10, IFN-γ, and TNF-α production by CD4^+^ T cells from naïve mice. Hence, these results provide evidence for DC-derived IL-10 to be involved in the induction of IL-10-producing CD4^+^ Tr1 cells during *P. yoelii* infection of BALB/c mice.

Since DC-specific IL-10 ablation in *P. yoelii*-infected mice interfered with the induction of IL-10-producing CD4^+^ Tr1 cells and resulted in elevated production of pro-inflammatory cytokines, we monitored progression of blood infection by microscopic examination of Giemsa-stained blood films. We did not detect any significant effect on parasite clearance (Figure 4C), confirming that the observed differences can be attributed directly to immune signaling and are not an indirect effect of lower parasite burden.

### *P. yoelii* Infection of DC-Specific IL-10-Deficient Mice Results in Elevated IL-12 Serum Levels

**Figure 5:**
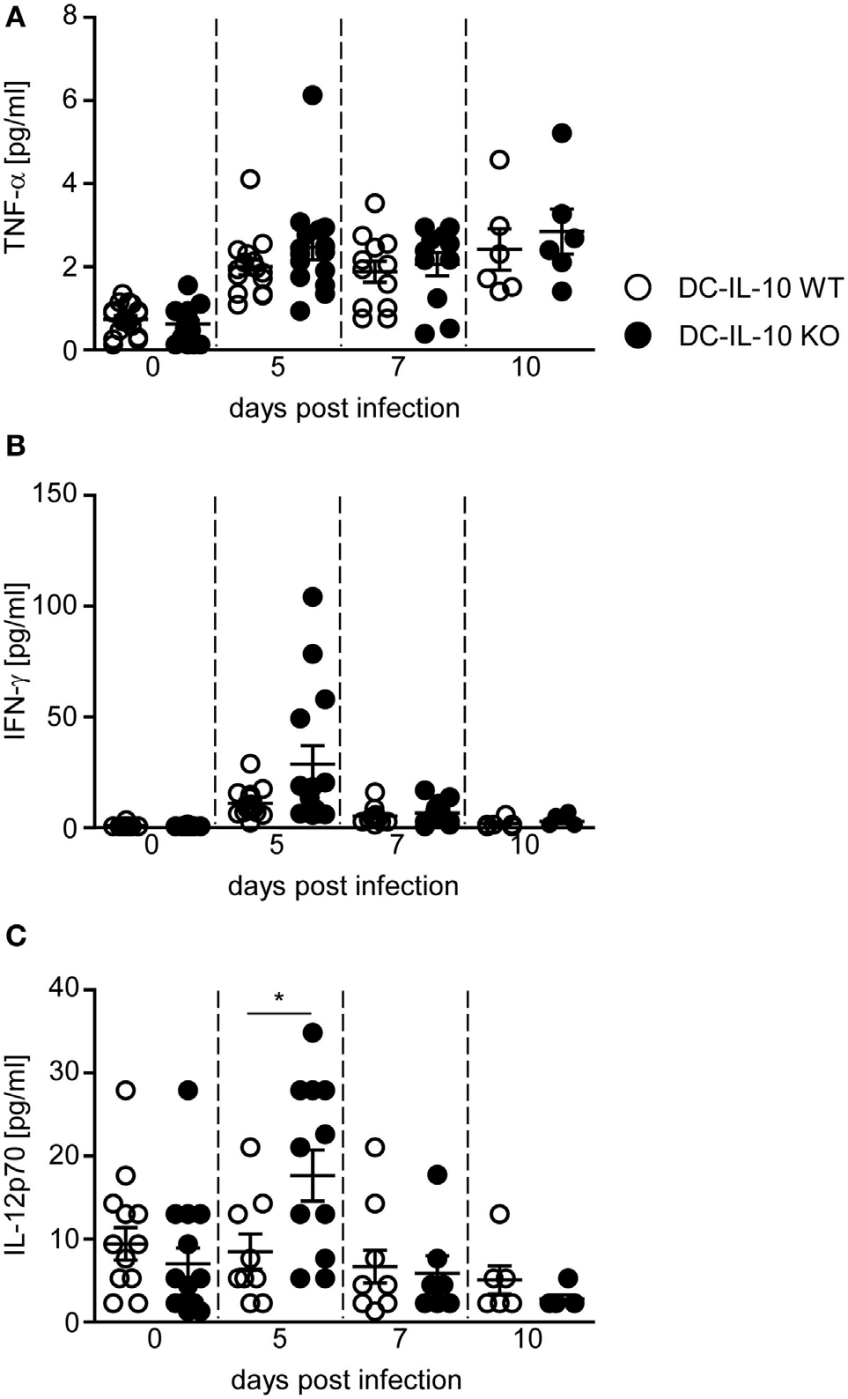
**IL-10 deficiency in CD11c^+^ dendritic cells (DCs) results in elevated IFN-γ and IL-12 serum level**. **(A)** TNF-α, **(B)** IFN-γ, and **(C)** IL-12 serum levels of *Plasmodium yoelii*-infected IL-10^flox/flox^ (DC-IL-10 WT) and IL-10^flox/flox^/CD11c-cre (DC-IL-10 KO) mice were determined by Luminex technology at indicated time points p.i. Results from at least two independent experiments with *n* = 6–12 mice per time point are summarized as mean ± SEM. Mann–Whitney test was used for statistical analysis (*p < 0.05).

Our results indicate that DC-specific ablation of IL-10 triggers the expression of pro-inflammatory cytokines in DCs and T cells, while dampening the induction of Tr1 cells during *P. yoelii* infection. To investigate whether these effects have also systemic implications, we analyzed the concentration of different cytokines in sera of *P. yoelii*-infected IL-10^flox/flox^/CD11c-cre mice and IL-10^flox/flox^ littermates. As depicted in Figure 5, DC-specific inactivation of IL-10 did not change TNF-α serum concentration (Figure 5A), but resulted in a slightly, albeit non-significantly increased systemic IFN-γ production (Figure 5B) and significantly elevated IL-12 levels (Figure 5C) in sera of *P. yoelii*-infected mice at day 5 postinfection.

## Discussion

The cytokine IL-10 has been identified as a key regulator to keep the balance between pro- and anti-inflammatory responses during the blood stage of *Plasmodium* infection. IL-10 deficiency resulted in a more efficient parasitic clearance upon *P. yoelii* infection and increased mortality of normally avirulent *P. chabaudi* infection (9, 33). Although IL-10 is a secreted cytokine that can have systemic effects, its cellular source appears to profoundly affect the resulting immune responses (34). We and others identified CD4^+^ T cells to produce high amounts of IL-10 and exhibiting suppressive activity during *Plasmodium* infection (3, 9, 21). However, it remains unclear whether induction of IL-10^+^ Tr1 cells results from direct interaction with the parasite or as a consequence of CD4^+^ T cell activation in the presence of IL-10; provided by professional APCs such as DCs, B cells, or macrophages (35).

Here, we demonstrate that the frequency of CD11c^+^CD11b^+^CD8^−^ IL-10-producing DCs was significantly elevated upon *P. yoelii* infection of BALB/c mice. Well in line with our results, an increase in IL-10 expression was also observed in CD11c^high^ DCs from *P. chabaudi*-infected and *P. berghei*-infected C57BL/6 mice (21, 22). However, to our knowledge, the role of DC-derived IL-10 during *Plasmodium* infection has not been analyzed in detail. Cell type-specific inactivation of IL-10 in CD11c^+^ DCs by using IL-10^flox/flox^/CD11c-cre mice resulted in higher frequencies of IFN-γ- and TNF-α-producing DCs (Figures 2C,D) accompanied by elevated IFN-γ production in CD4^+^ T cells (Figure 3B) from *P. yoelii*-infected mice. Additionally, our study revealed that DC-specific inactivation of IL-10 dampens the percentage of IL-10-expressing immunosuppressive CD4^+^ Tr1 cells at day 5 and 7 postinfection (Figure 4B). Therefore, we postulate that DC-derived IL-10 rather than direct interaction with the parasite promotes IL-10 production in CD4^+^ T cells upon *P. yoelii* infection. During *P. berghei* infection of C57BL/6 mice B cell-derived IL-10 was proposed to be involved in the induction of IL-10-producing CD4^+^ Tr1 cells. Coculture of purified IL-10^+^ B cells, but not IL-10^−^ B cells isolated from *P. berghei*-infected mice induced IL-10 production by CD4^+^ T cells (22) and B cell-specific inactivation of IL-10 lowered the number of IL-10 secreting CD4^+^ T cells during *P. chabaudi* infection (21). In our experiments, we have not detected a significant increase of IL-10 expression in CD19^+^ B cells or CD19^−^CD138^+^ plasma cells in the course of *P. yoelii* infection (Figures S5A,B in Supplementary Material). Hence, it seems to be unlikely that B cell-derived IL-10 contributes to elevated IL-10 expression in CD4^+^ T cells from *P. yoelii*-infected BALB/c mice. However, we cannot formally exclude that IL-10 produced by other cell types might also have an impact on the induction of CD4^+^ Tr1 cells during *P. yoelii* infection of BALB/c mice.

Analysis of systemic cytokine levels revealed increased IFN-γ and IL-12 serum concentrations at day 5 post-*P. yoelii* infection in IL-10^flox/flox^/CD11c-cre mice compared to WT mice, suggesting that IL-10 produced by either DCs or CD4^+^ T cell or both immune cell subsets modulates the production of circulating pro-inflammatory cytokines. Well in line, significantly higher plasma levels of IFN-γ and IL-12 have also been observed in *P. chabaudi*-infected IL-10^−/−^ mice (33) underlining the importance of IL-10 in regulating pro-inflammatory immune responses during *Plasmodium* infection.

Our results indicate that the parasite *P. yoelii* promotes IL-10 production in CD11c^+^CD11b^+^CD8^−^ DCs, which in turn induces IL-10-expressing Tr1 cells. Evidence for parasite-induced IL-10 expression in DCs due to direct interaction was provided by coculture experiments of DCs with infected RBCs resulting in elevated IL-10 expression (36, 37). However, it remains unclear by which mechanism the parasite stimulates IL-10 production in DCs. Previous studies proposed that heme, which is released after lysis of iRBCs or hemozoin, the product of hemoglobin degradation by *Plasmodium* parasites is involved in the induction of IL-10 in murine and human macrophages (38, 39) and resulted in downregulation of IL-12 in human PBMCs and CD14^+^ monocytes (40). Hemozoin (41) or a complex of hemozoin, DNA, and unknown proteins (42) have been described to bind and trigger toll-like receptor 9 signaling. In addition, intact *Plasmodium falciparum*-infected erythrocytes can bind directly to CD36 and treatment of human DCs with anti-CD36 resulted in elevated IL-10 expression to comparable levels as after treatment with iRBCs (36). However, which receptors and signaling pathways are involved in the induction of IL-10 expression in CD11c^+^CD11b^+^CD8^−^ DCs during *P. yoelii* infection has to be elucidated in further experiments.

Interestingly, our data show that DC-derived IL-10 has no impact on pathogen clearance, although we detected elevated cellular and systemic pro-inflammatory cytokine levels and lower percentages of IL-10-producing CD4^+^ Tr1 cells in *P. yoelii*-infected IL-10^flox/flox^/CD11c-cre mice than in respective control animals. T cell-specific inactivation of IL-10 had also no impact on parasitemia in *P. yoelii*-infected BALB/c mice in our hands (3) and on parasite burden in C57BL/6 mice infected with *P. chabaudi* (21). In contrast, Couper and colleagues reported a significant influence of T cell-derived IL-10 on parasite clearance. The apparent discrepancy can likely be attributed to the different experimental settings, parasite propagation in T cell-specific IL-10-deficient mice (3, 21) and adoptive transfer of CD4^+^ T cells from IL-10-deficient C57BL/6 to *P. yoelii*-infected Rag1KO mice, which also lack CD8^+^ T cells and B cells, in addition to endogenous CD4^+^ T cells (9).

In summary, our results provide evidence that *P. yoelii* infection of BALB/c mice results in elevated IL-10 expression in CD11c^+^CD11b^+^CD8^−^ DCs, which in turn dampens the cellular and systemic production of pro-inflammatory cytokines and induces IL-10-secreting CD4^+^ Tr1 cells. These results contribute to a better understanding in the impact of DC-derived IL-10 on the dysregulated immune responses during *Plasmodium* infection.

## Author Contributions

KL, KU, SA, and MH designed and performed the experiments and analyzed data. JB, AW, and KM were involved in the data discussion and in drafting the manuscript. WH initiated, organized, and designed the study and wrote the manuscript.

## Conflict of Interest Statement

The authors declare that the research was conducted in the absence of any commercial or financial relationships that could be construed as a potential conflict of interest.

## Abbreviations

APC, antigen-presenting cell; DC, dendritic cell; iRBC, infected red blood cells; *P. yoelii, Plasmodium yoelii*; Tr1, type 1 regulatory T cell; Treg, regulatory T cell.
