# Supplementary material for: DC-Derived IL-10 Modulates Pro-inflammatory Cytokine Production and Promotes Induction of CD4+IL-10+ Regulatory T Cells during Plasmodium yoelii Infection
Source: Front Immunol. 2017 Feb 28;8:152. doi: 10.3389/fimmu.2017.00152 (PMC5328999; doi:10.3389/fimmu.2017.00152)
Supplement: Supplementary file 1 [file Presentation_1.PDF]

Figure S1, Loevenich et al.

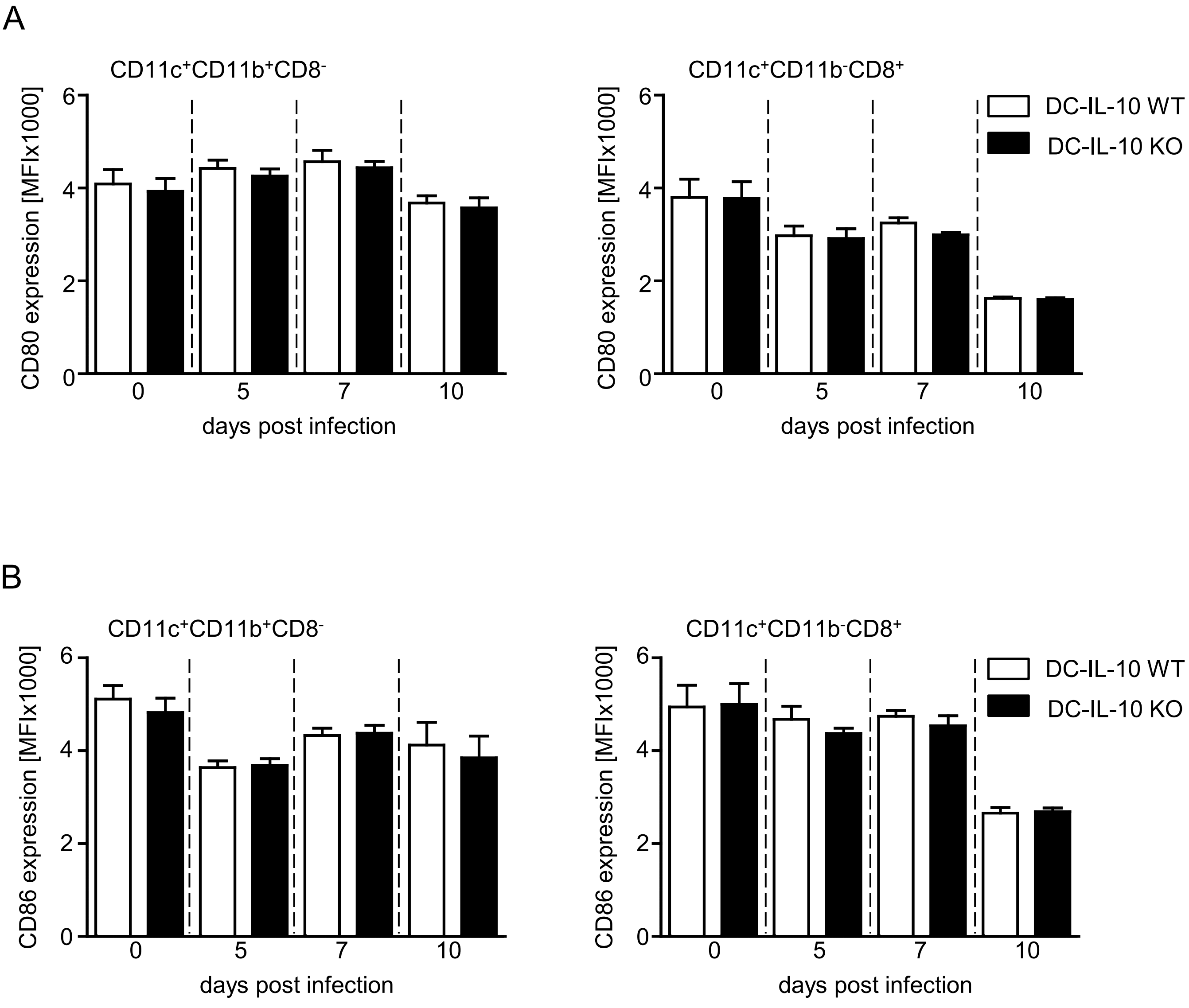

FIGURE S1. CD80 and CD86 expression on dendritic cells from *P. yoelii*-infected DC-specific IL-10-deficient and WT mice. (A) CD80 and (B) CD86 expression levels (MFI) on CD11c$^+$CD11b$^+$CD8$^-$ DCs (left panel) and CD11c$^+$CD11b$^-$CD8$^+$ DCs (right panel) were analyzed in non-infected and *P. yoelii*-infected IL-10$^{flox/flox}$ (DC-IL-10 WT) and IL-10$^{flox/flox}$/CD11c-cre (DC-IL-10 KO) mice at day 5, 7, and 10 p.i. by flow cytometry. Results from at least two independent experiments with n = 6 – 16 mice per time point were summarized as mean ± SEM.

Figure S2, Loevenich et al.

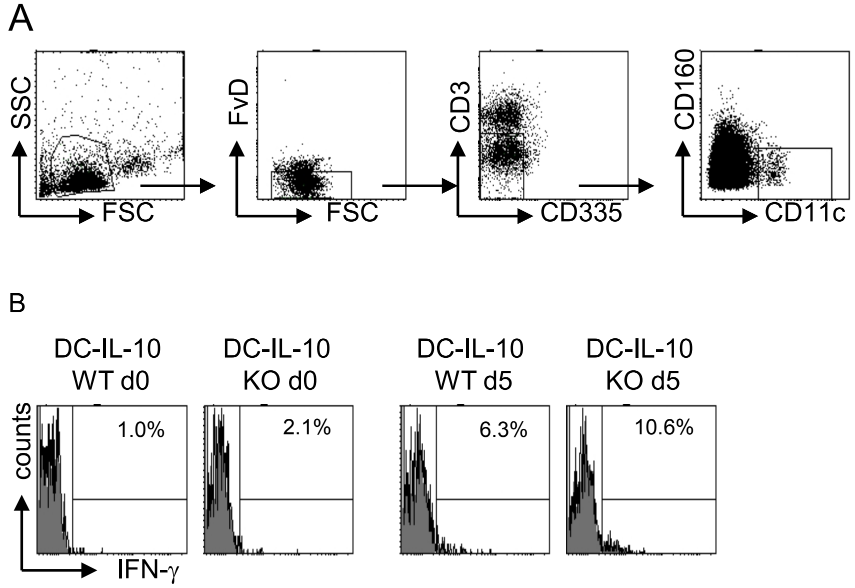

FIGURE S2. IFN-γ expression of dendritic cells from *P. yoelii*-infected DC-specific IL-10-deficient and WT mice. (A) Gating strategy: mononuclear cells were selected (FSC/SSC) and dead cells were excluded by using an APC-Cy7-coupled fixable viability dye (FvD). $CD3^{-}CD335^{-}$ cells were selected, before gating on $CD11c^{+}CD160^{-}$ dendritic cells. (B) The percentage of IFN-γ expressing $CD3^{-}CD335^{-}CD160^{-}CD11c^{+}$ DCs was analyzed in non-infected and *P. yoelii*-infected IL-$10^{flox/flox}$ (DC-IL-10 WT) and IL-$10^{flox/flox}$/CD11c-cre (DC-IL-10 KO) mice at day 5 p.i. by flow cytometry. Representative histograms from two independent experiments with n = 4 – 8 mice are shown.

Figure S3, Loevenich et al.

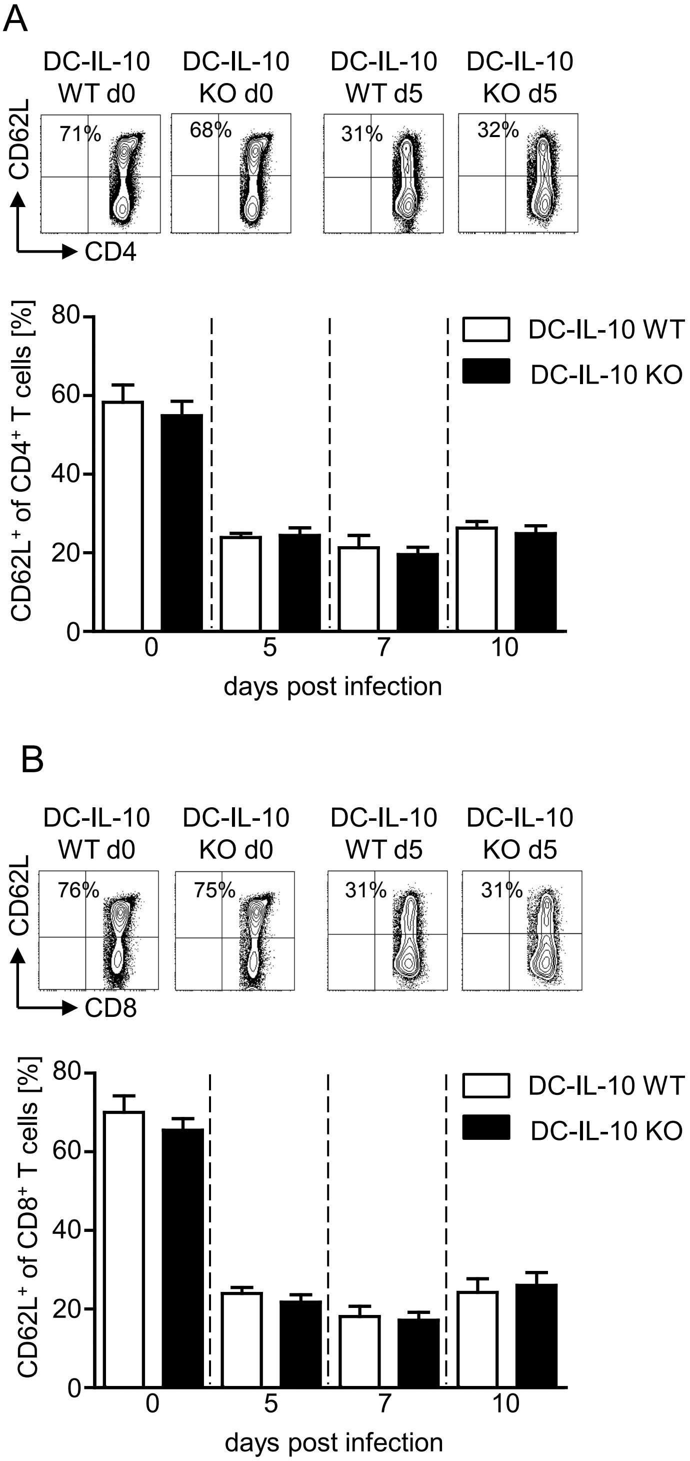

FIGURE S3. Inactivation of IL-10 in DCs has no influence on CD62L expression of T cells from *P. yoelii*-infected mice. IL-$10^{flox/flox}$ (DC-IL-10 WT) and IL-$10^{flox/flox}$/CD11c-cre (DC-IL-10 KO) mice were infected with *P. yoelii*. At different time points post infection the frequency of CD62L-expressing (A) $CD4^+$ and (B) $CD8^+$ T cells was determined by flow cytometry. Representative dot plots are depicted in the upper panels. Results from at least two independent experiments with n = 6 – 16 mice per time point were summarized as mean ± SEM.

Figure S4, Loevenich et al.

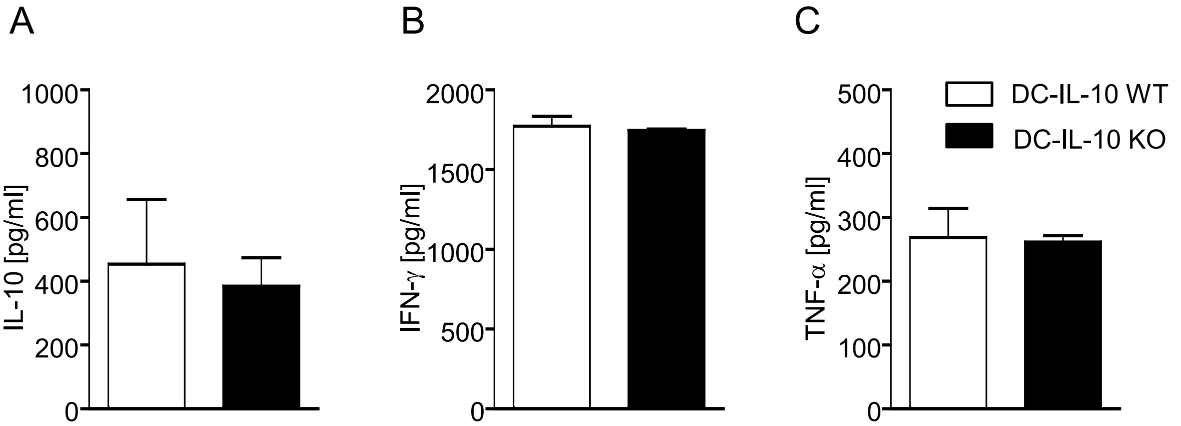

FIGURE S4. CD4+ T cells from naïve IL-10$^{flox/flox}$/CD11c-cre and IL-10$^{flox/flox}$ control mice do not differ in their cytokine production after stimulation *in vitro*. CD4+ T splenocytes were sorted from IL-10$^{flox/flox}$ (DC-IL-10 WT) and IL-10$^{flox/flox}$/CD11c-cre (DC-IL-10 KO) mice and stimulated with anti-CD3/anti-CD28 for 72h *in vitro*. The amount of secreted (A) IL-10, (B) IFN-γ and (C) TNF-α was determined in the supernatants by Luminex technology. Results from two independent experiments with n = 3 – 5 mice were summarized as mean ± SEM.

Figure S5, Loevenich et al.

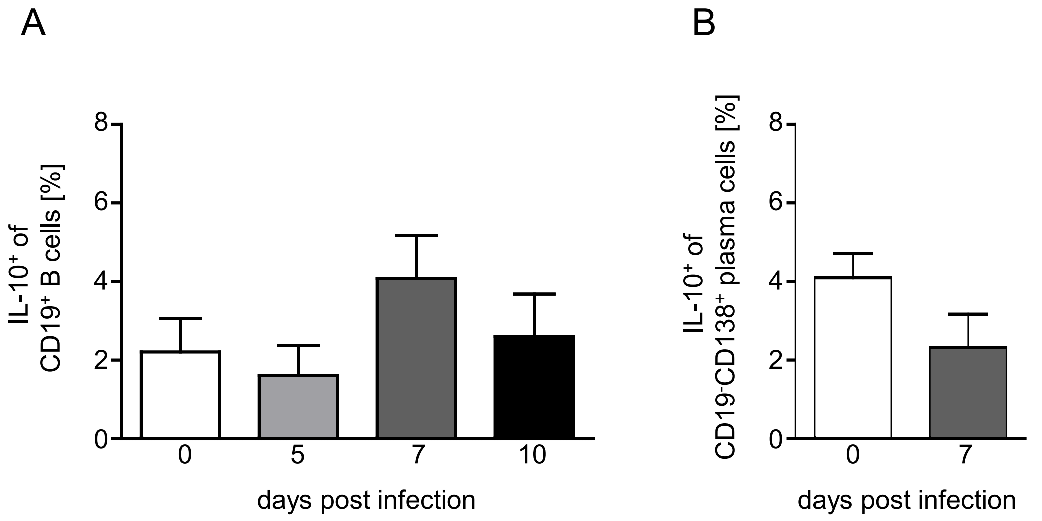

FIGURE S5. IL-10 production of $CD19^+$ B cells and $CD19^-CD138^+$ plasma cells during *P. yoelii* infection. The percentage of IL-10 producing (A) $CD19^+$ B cells and (B) $CD19^-CD138^+$ plasma cells was analyzed in *P. yoelii*-infected IL-10eGFP reporter mice at indicated time points p.i.. Results from at least two independent experiments with n = 7 – 11 mice ($CD19^+$ B cells) or n = 3 – 5 mice ($CD19^-CD138^+$ plasma cells) per time point were summarized as mean ± SEM.
